# Unraveling diffusion behavior in Cu-to-Cu direct bonding with metal passivation layers

**DOI:** 10.1038/s41598-024-57379-2

**Published:** 2024-03-20

**Authors:** Min Seong Jeong, Sang Woo Park, Yeon Ju Kim, Ji Hun Kim, Seul Ki Hong, Sarah Eunkyung Kim, Jong Kyung Park

**Affiliations:** Department of Semiconductor Engineering, Seoul National University of Science and Technology, Seoul, Republic of Korea

**Keywords:** Engineering, Chemical engineering

## Abstract

Cu/SiO_2_ hybrid bonding presents a promising avenue for achieving high-density interconnects by obviating the need for microbumps and underfills. Traditional copper bonding methods often demand temperatures exceeding 400 °C, prompting recent endeavors to mitigate bonding temperatures through investigations into metal passivation bonding. In this study, we scrutinized the diffusion behavior associated with various metal passivation layers (Platinum, Titanium, Tantalum, and Chromium) in the context of low-temperature direct copper bonding and delved into the essential bonding mechanisms. We observed a deviation from conventional metal–metal bonding factors, such as surface roughness and grain size, in the diffusion behavior. Remarkably, our analysis revealed a pronounced correlation between the crystallinity of the metal passivation layers and diffusion behavior, surpassing the influence of other experimental factors. Subsequent post-bonding examinations corroborated consistent diffusion behavior in Pt and Cr passivation samples with disparate crystallinities, reinforcing the significance of crystallinity in the bonding process. Our findings underscore crystallinity as a pivotal factor governing diffusion behavior, even under varied bonding conditions. These insights are instrumental in achieving exceptional bonding characteristics at lower temperatures in Cu/SiO_2_ hybrid bonding. Implications of this study extend to the prospect of advancing highly integrated systems through die-to-wafer bonding, marking a substantial stride toward future applications.

## Introduction

The performance of semiconductors has primarily been achieved through the miniaturization of devices. As devices are miniaturized to the scale of a few nanometers, economic and physical limits are being reached. As the miniaturization of devices, following Moore's Law, approaches its limits, efforts are ongoing to enhance the performance of devices through 3D packaging technologies. Efforts for miniaturization continue, but with the emerge of advanced packaging technologies, performance enhancement through packaging is gaining even more attention.

As the I/O density increases, packaging methods using solder bumps can pose reliability issues. Additionally, there is another issue of increased resistance due to the formation of Inter-Metallic Compound (IMC), leading to decreased efficiency. Therefore, direct bonding of signal transmission lines using Cu is considered important. Furthermore, hybrid bonding technology that simultaneously bonds Cu and dielectrics is also gaining attention^1^. While Cu is primarily used for signal transmission lines due to its excellent electrical properties, the direct bonding process of Cu faces many challenges, mainly because Cu readily forms a native oxide layer. Copper bonding with a naturally formed oxide layer requires a temperature of 400 °C^2^, and such high-temperature processes can lead to a degradation in the performance of devices. Therefore, there has been extensive research aimed at overcoming this challenge. The Surface Activated Bonding (SAB) process, which involves activating the copper surface in Ultrahigh Vacuum, has a significant advantage of being a room temperature process^3^. However, it faces the challenge of being expensive for mass production due to the bonding process taking place in a high vacuum environment. There has been research introducing the use of wet chemicals to remove oxides, facilitating bonding^4^. This method also has challenge, including the potential for residual contaminants and the possibility of performance degradation during the treatment process. Another method involves the application of Self-Assembled Monolayer (SAM) to inhibit natural oxidation and facilitate bonding^5^. This method also has the challenge of potentially leaving residual contaminants and requiring additional heat treatment to remove the monolayer. In addition, various research is being conducted, such as using plasma to inhibit native oxide^6,7^ and altering material properties by controlling the crystal orientation of copper^8^. Among these efforts, research utilizing a metal passivation layer on top of a copper layer is actively being conducted^9–11^. The metal passivation layer is a method similar to using SAM, inhibiting native oxide and enabling copper bonding. This method has the advantage of enabling copper bonding at relatively low temperatures and being free from residual contamination. Using the Atomic Layer Deposition (ALD) deposition method, there is considerable interest in research focused on selectively depositing passivation layers on specific copper area^12,13^. While this process method is advantageous for application in hybrid bonding, it has the challenge of relatively high deposition temperatures and the use of oxygen as a reactant, which raises concerns about oxidation.

Previously, a variety of passivation materials, including Au, Cr/Au, Pd, Ag, Pt, and Ti^9–11,14–17^, have been utilized. However, these materials have been primarily investigated solely for their role in enhancing bonding quality and electrical properties within homogeneous materials. Therefore, rather than concentrating solely on low-temperature metal passivation bonding to enhance bonding performance, this study aims to elucidate the underlying reasons for variances in diffusion behavior observed depending on the metal passivation material. In other words, the primary focus of this paper is to identify the material characteristics and bonding process that exert the most significant influence on Cu-Cu diffusion bonding. In this study, various metal materials were used to compare the differences in diffusion behavior. Noble metal Pt with good Cu diffusion^15^, Ti that has been studied a lot with excellent diffusion capability^16^, Ta that is also used as a diffusion barrier^18^, and Cr that is also used as a wetting layer because of its smooth roughness^14,17^ were selected. Pt and Ta materials were chosen as exemplary candidates for highlighting disparities between materials with robust diffusion and those with limited diffusion. It is more advantageous for comparison purposes to include materials exhibiting poor diffusion rather than encompassing solely those demonstrating diffusion. This is because identifying the characteristics of materials with limited diffusion and contrasting them with other materials holds paramount importance. This material selection is considered suitable for unraveling the cause of the diffusion difference by comparing materials with various diffusion properties. When selecting passivation materials in much research, there are many options to consider, including cost, suitability for the desired devices, and diffusion behavior based on film characteristics. By comparing and adding factors influencing diffusion behavior, it is expected that this research will provide important guidelines for selecting passivation materials.

## Methods

### Sample preparation

**Figure 1:**
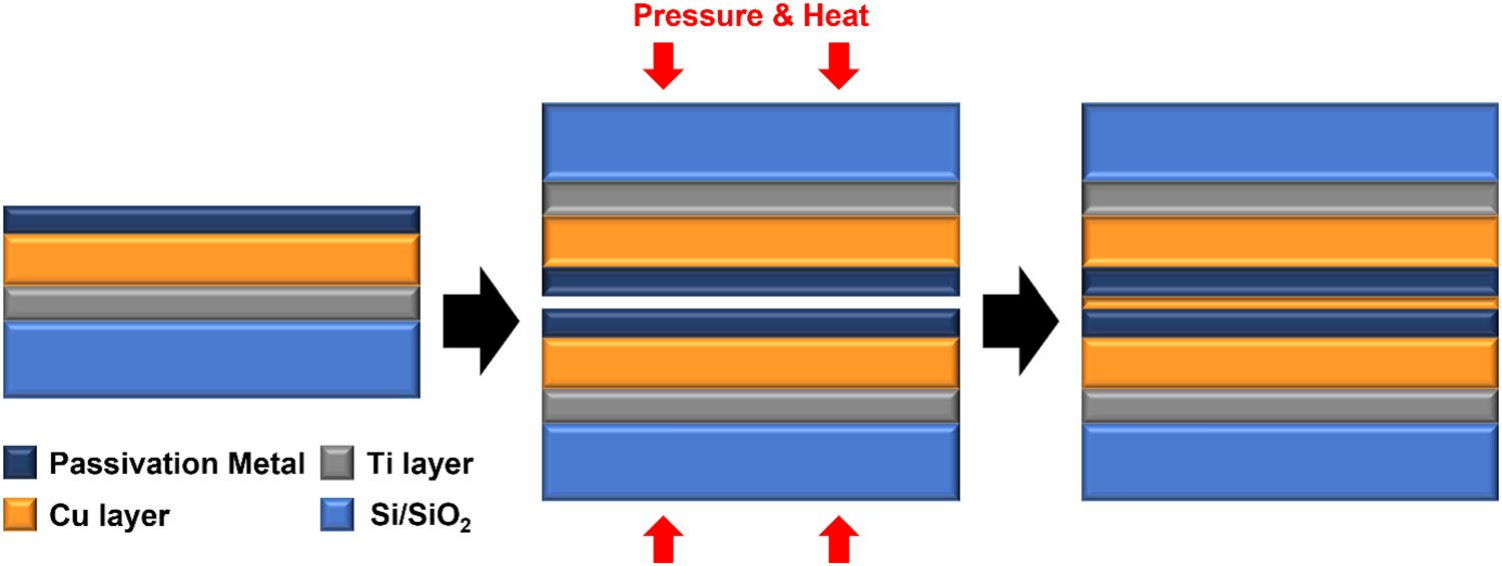
Schematic of deposition sequence and the process steps used in the bonding experiment.

An 8-inch silicon wafer is subjected to thermal oxidation to form a 700 nm SiO_2_ layer. To facilitate deposition in pieces, it is diced into 1 × 1 cm^2^ using an Automatic Dicing Saw System (DAD3350, DHK SOLUTION Corp.). The diced sample was deposited using Metal Cluster Sputter (SME-200 J, ULVAC). Titanium (50 nm) was first deposited as the adhesion layer, followed by Copper (1 μm). Subsequently, the passivation layer, consisting of Pt, Ti, Ta and Cr, was deposited. Each passivation layer was deposited to a thickness of 10–12 nm, and all metal layers, including the adhesion Ti layer, were processed in-situ in a high vacuum chamber environment without venting to prevent native oxide. The thickness was selected as the thickness adopted by many researchers. Furthermore, it was selected to minimize errors, as significant errors can occur during the sputtering process for sub-nanometer passivation layers. The deposition sequence and the bonding process utilized in the experiment are illustrated in Fig. 1.

### Sample analysis and bonding experiment

After deposition, the samples were subjected to depth profile analysis using a High-performance X-ray Photoelectron Spectrometer (Thermo Fisher Scientific, HPXPS) to investigate diffusion behavior. Prior to the analysis, an annealing process was conducted at 200 °C for 1 h. Furthermore, to compare inter-diffusion and activation energies, additional analysis was conducted on samples annealed for 1 h at various temperatures (25/150/200 °C) using X-ray photoelectron spectroscopy (NEXSA, ThermoFisher Scientific, XPS). Surface roughness and morphology were analyzed using Atomic Force Microscopy (XE100/XE150, PSIA, AFM). Additionally, to obtain information regarding grain size and crystallinity, analysis was conducted using a Multi-Purpose X-Ray Diffractometer (X'Pert Pro MPD, PANalytical, XRD). AFM and XRD analyses were conducted without additional processing after deposition. After the bonding process, Transmission Electron Microscope (JEM-2100F, JEOL, HR-TEM) analysis was conducted to compare the diffusion behavior at the interface. The bonding samples with a metal passivation layer were bonded using a High Accuracy Flip-Chip Bonder (Accura opto 100, SET, Flip-Chip Bonder) under various conditions. Also, to observe differences in the bonding quality of the samples, Scanning Acoustic Microscopy (FineSAT, HITACHI, SAM) was performed.

## Results and discussion

**Figure 2:**
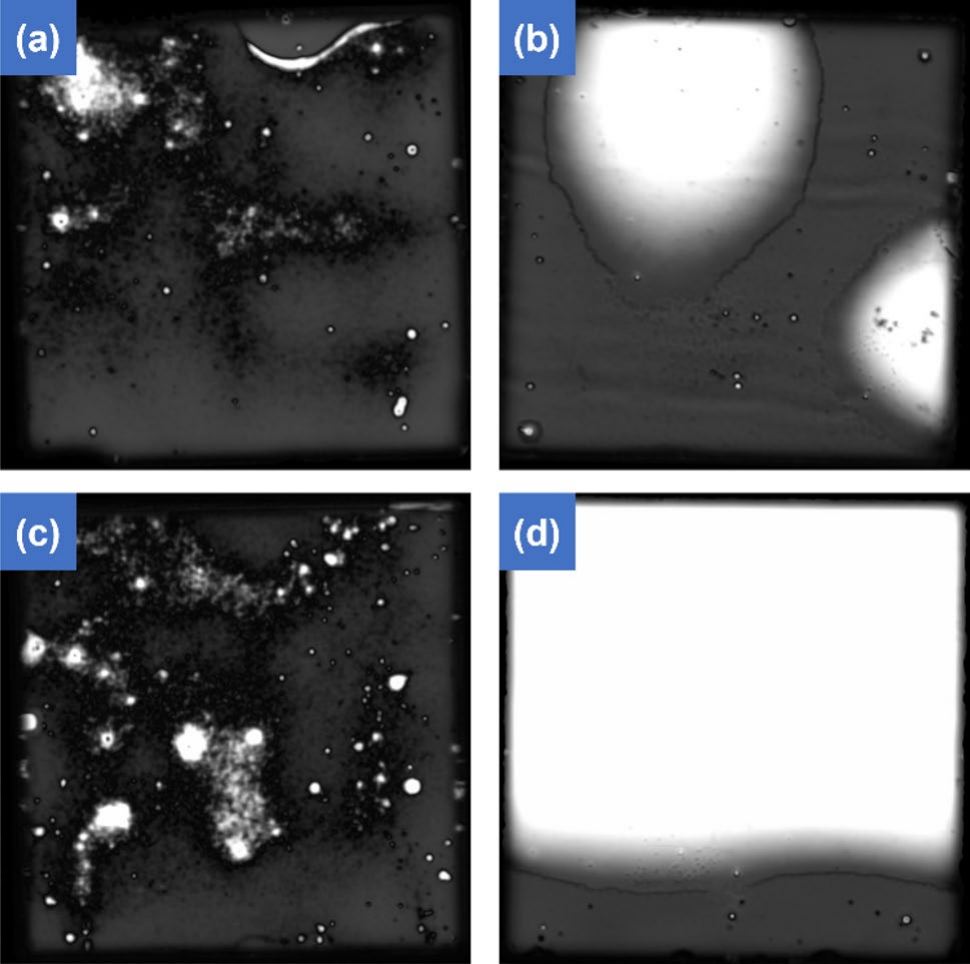
SAM images of bonding samples. (**a**) Pt and (**b**) Cr passivated sample at 280 °C, 1 h and 4.5 MPa condition and (**c**) Pt and (**d**) Cr passivated sample at 260 °C, 1 h condition and 4.5 MPa.

Figure 2 is a SAM image confirming the interface between the samples bonded with Pt and Cr layers on top of the Cu layer. Although the thickness of Pt and Cr are same, when inspecting the SAM image, they exhibit different interfaces. The black areas represent the bonded regions, whereas the white areas indicate areas where did not bonding. Non-bonded areas can be made for reasons such as particles or contamination, resulting in voids. As all bonding processes were conducted in ambient environment rather than a vacuum, numerous voids were observed. In the case of the sample using Pt Fig. 2a, almost all areas exhibit successful bonding, while for the sample with Cr applied Fig. 2b, many areas show a lack of bonding. For the sample with Pt applied Fig. 2c, it can be observed that bonding occurred at a similar level even though the temperature was slightly lower. However, for the sample using Cr Fig. 2d, a significant decrease in the level of bonding can be observed. Also, samples using Ti and Ta were bonded under the same conditions as Pt, but the bonding level was significantly decreased. Images and detail description are attached as Fig. S1a,b. To identify the reason for the observed differences despite the same thickness of applied metal passivation and bonding conditions, performed XPS analysis.

### Comparison of diffusion behavior using XPS

**Figure 3:**
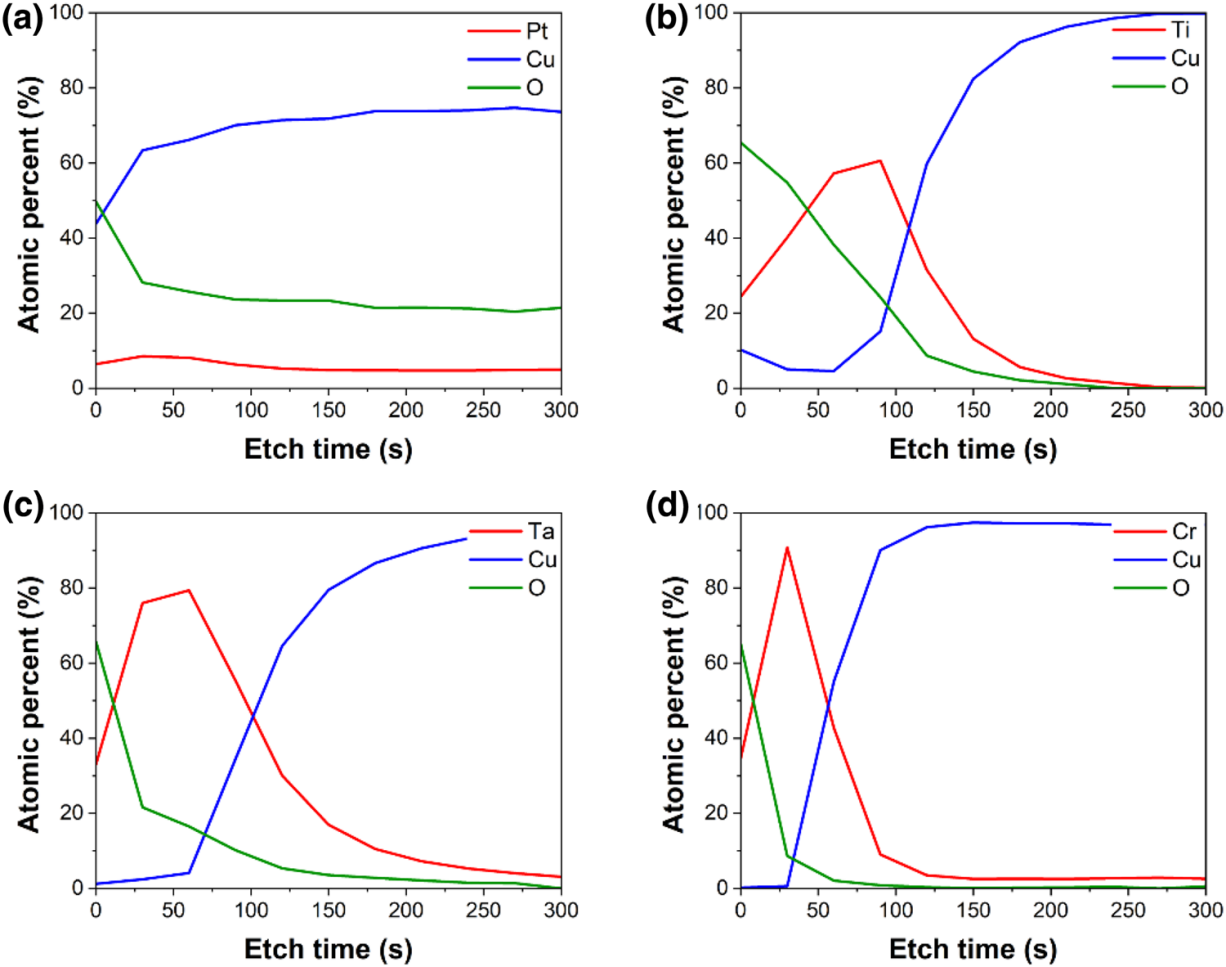
XPS depth profiles to confirmed diffusion behavior (after annealing at 200 °C, 1 h). (**a**) Pt, (**b**) Ti, (**c**) Ta, (**d**) Cr passivated sample.

The diffusion behavior of samples with various metal layers applied on top of the Cu were investigated using XPS depth profile. Figure 3 shows the diffusion behavior of samples annealed at 200 °C for 1 h. The rationale behind selecting a temperature of 200 °C for XPS analysis, which is lower than the bonding temperature, is that when samples undergo heat treatment at a higher temperature of 280 °C, they exhibit a uniform diffusion behavior, thereby complicating the differentiation of diffusion variances. This phenomenon is attributed to the distinction between the bonding conditions and the actual environment utilized for XPS sample analysis. Consequently, we substantiated the interface analysis results through TEM after bonding at 200 °C, as illustrated in Fig. 8. As observed in graph Fig. 3a, the sample with Pt applied shows relatively uniform presence of the Pt layer from the surface to the interior, indicating good inter-diffusion between the Cu layer and passivation layer. However, for the other samples, the applied metal passivation layers show relatively less diffusion, with a significant presence near the surface. The sample using Pt metal Fig. 3a exhibits the highest amount of Cu diffusion at the surface (etch level 0), reaching 43.9%. Subsequently, for Fig. 3b Ti, it decreased to 10.2%, Fig. 3c Ta to 1.2%, and Fig. 3d Cr to 0.2%. Consistent with the trends observed in the mentioned graphs, for Pt, there is good inter-diffusion resulting in a significant amount of Cu diffusion to the surface. In contrast, for the other cases, the diffusion is less exhibited, meaning to a relatively lower amount of Cu diffusion to the surface. Especially in the case of Cr, it is noticeable that there is a substantial amount of Cr near the surface, and the diffused Cu is the least among all cases.

**Figure 4:**
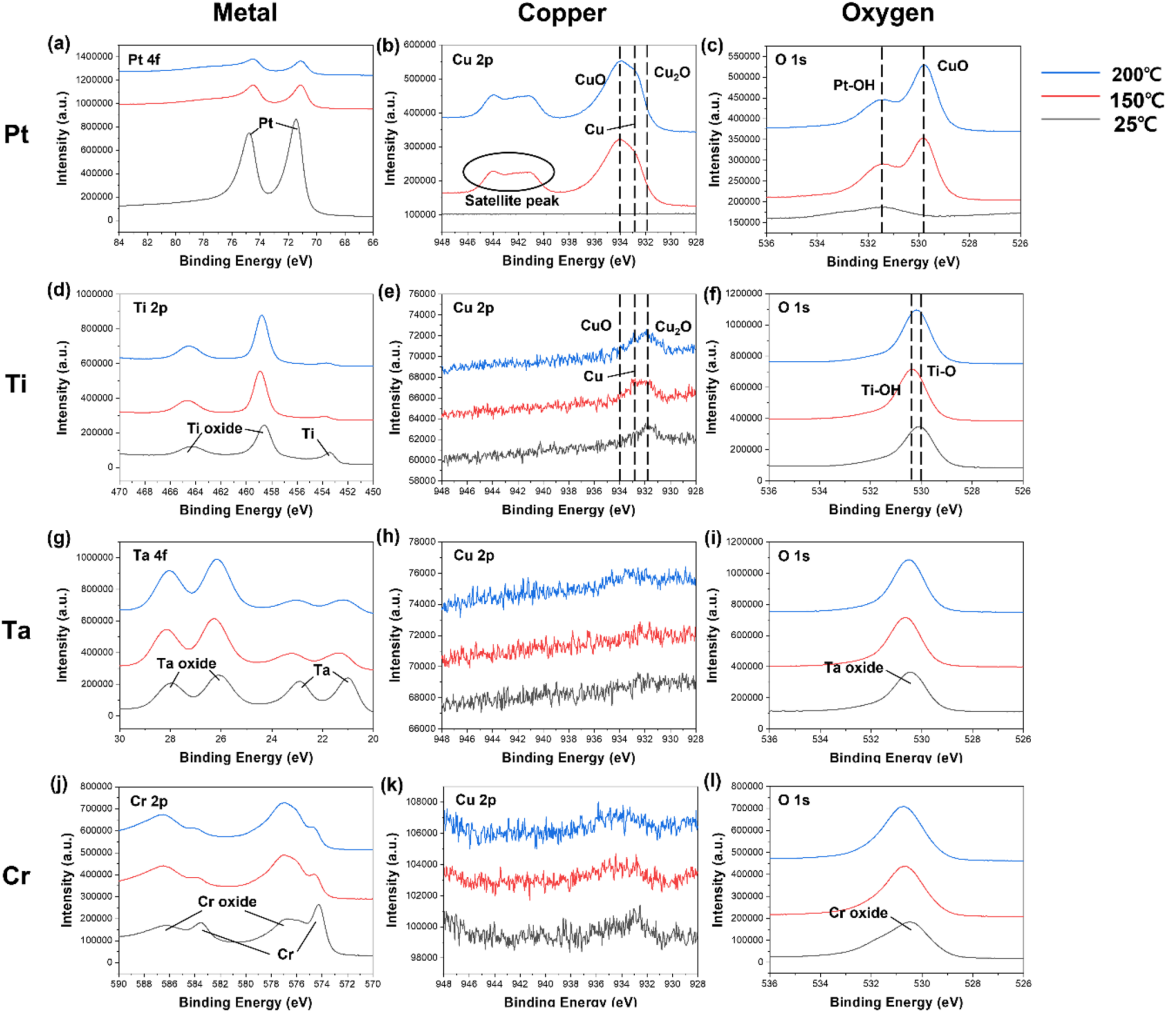
Surface chemical bonds XPS graphs using various metal passivation. (**a**–**c**) Pt, (**d**–**f**) Ti, (**g**–**i**) Ta and (**j**–**l**) Cr sample. Each metal contains metal, Cu and O peak.

Following the diffusion behavior observed in the XPS depth profile, surface XPS analysis of samples annealed at various temperatures was conducted to investigate the inter-diffusion between metal and Cu. The XPS graphs are shown in Fig. 4. In the case of Fig. 4a,d, it may be observed that as the temperature increases, the metal on the surface diffuses to the lower Cu, and thus the peak decreases. Accordingly, as the Cu of the lower layer inter-diffused, Cu peaks were confirmed as shown in Fig. 4b,e. It was observed that the oxidation of diffused Cu proceeded during annealing process. In Fig. 4b, Pt sample has lot of diffused Cu, satellite peaks present as Cu^2+^ and Cu^+^ on the surface are also observed. Peak of Fig. 4c,f, CuO and Ti–O bonding are mainly identified, respectively. In Fig. 4d,g,j, it was observed that the metal peak decreased as the temperature increased, simultaneously, oxidation proceeded to increase the oxide peak. But Fig. 4h,k, it was difficult to confirm the peak because there was no diffused Cu. Accordingly, oxide peaks were observed in Fig. 4i,l.

In the low-temperature diffusion process, the grain boundary diffusion coefficients were calculated using the Modified Surface Accumulation method through the property that grain boundary diffusion predominates^19,20^. Table 1 show the diffusion coefficients and experimental conditions for the passivation materials. The result confirmed that the diffusion coefficient decreased in the order of Pt, Ta, and Cr. Ti sample could not confirm the factor required for calculating the diffusion coefficient in the XRD graph (refer to Fig. S3 and XRD part). In addition, the activation energy was compared after the annealing process at different temperatures, with various passivation applied samples^21,22^ (Fig. S2). Even in annealing process, there were samples without Cu diffused over the surface. Since it proceeds through inter-diffusion rather than single-layer diffusion, it was compared through metal into Cu instead of Cu into metal. The Pt sample showed the lowest activation energy. However, for the other samples, they showed almost negligible differences among each other. But there was a considerable difference from Pt. This result is similar with the trend observed in the XPS depth profile. In the Pt sample, inter-diffusion was best seen in the XPS diffusion behavior, and the activation energy also showed the smallest value. Various analyses were conducted to find out the cause of the difference in diffusion behavior identified as above.

**Table 1:** Summarize copper into metal diffusion coefficients.

Experimental conditions		Diffusion coefficient (m^2^/s)		
Temp. (°C)	Time (s)	Pt	Ta	Cr
200	3600	6.0 × 10^–21^	8.3 × 10^–23^	1.3 × 10^–23^

### Comparison of surface roughness for various metal passivation layers using AFM

**Figure 5:**
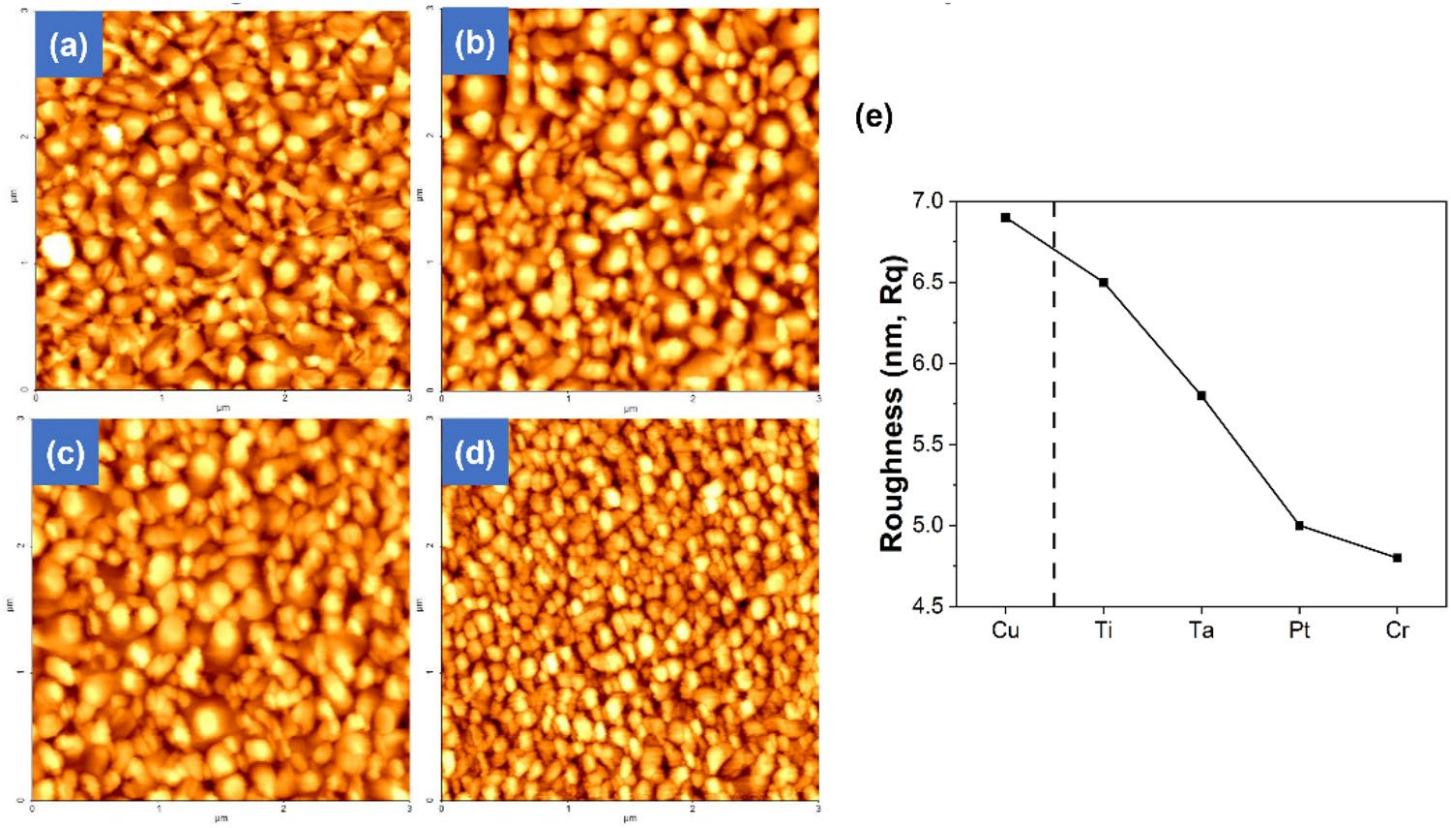
AFM images and graph to verification surface roughness and morphology. (**a**) Ti, (**b**) Ta, (**c**) Pt, (**d**) Cr passivated sample, (**e**) surface roughness values plotted graph.

Surface roughness and morphology of various metal passivation layers were examined using AFM. The surface roughness of the bare Cu layer without a metal passivation layer is 6.9 nm, indicating a rough surface. On the contrary, when using metal passivation layers, the surface roughness are observed to decrease compared to the bare Cu layer. The reduction in surface roughness at the bonding interface is known to minimize void formation by increasing the contact area, thereby influencing the bonding quality^23^. Figure 5 shows the surface roughness and morphology of various passivation layers applied on top of Cu. With values of Fig. 5a Ti passivation (6.5 nm), Fig. 5b Ta (6.0 nm), Fig. 5c Pt (5.8 nm), and Fig. 5d Cr (4.8 nm), applying the Cr passivation layer on the Cu layer resulted in the most significant reduction in surface roughness, with a decrease of 2.1 nm. However, when using Ti passivation, the reduction in surface roughness is the least, with a decrease of 0.4 nm. The confirmed surface roughnesses are presented in graph Fig. 5e. Considering only the surface roughness, sample with Cr application has the potential for the superior bonding quality. However, it can be noted that it shows a different trend from the previously observed diffusion behavior. Therefore, additional comparisons of other factors influencing diffusion behavior are necessary.

### Comparison of grain size and crystallinity for various metal passivation layers using XRD

**Figure 6:**
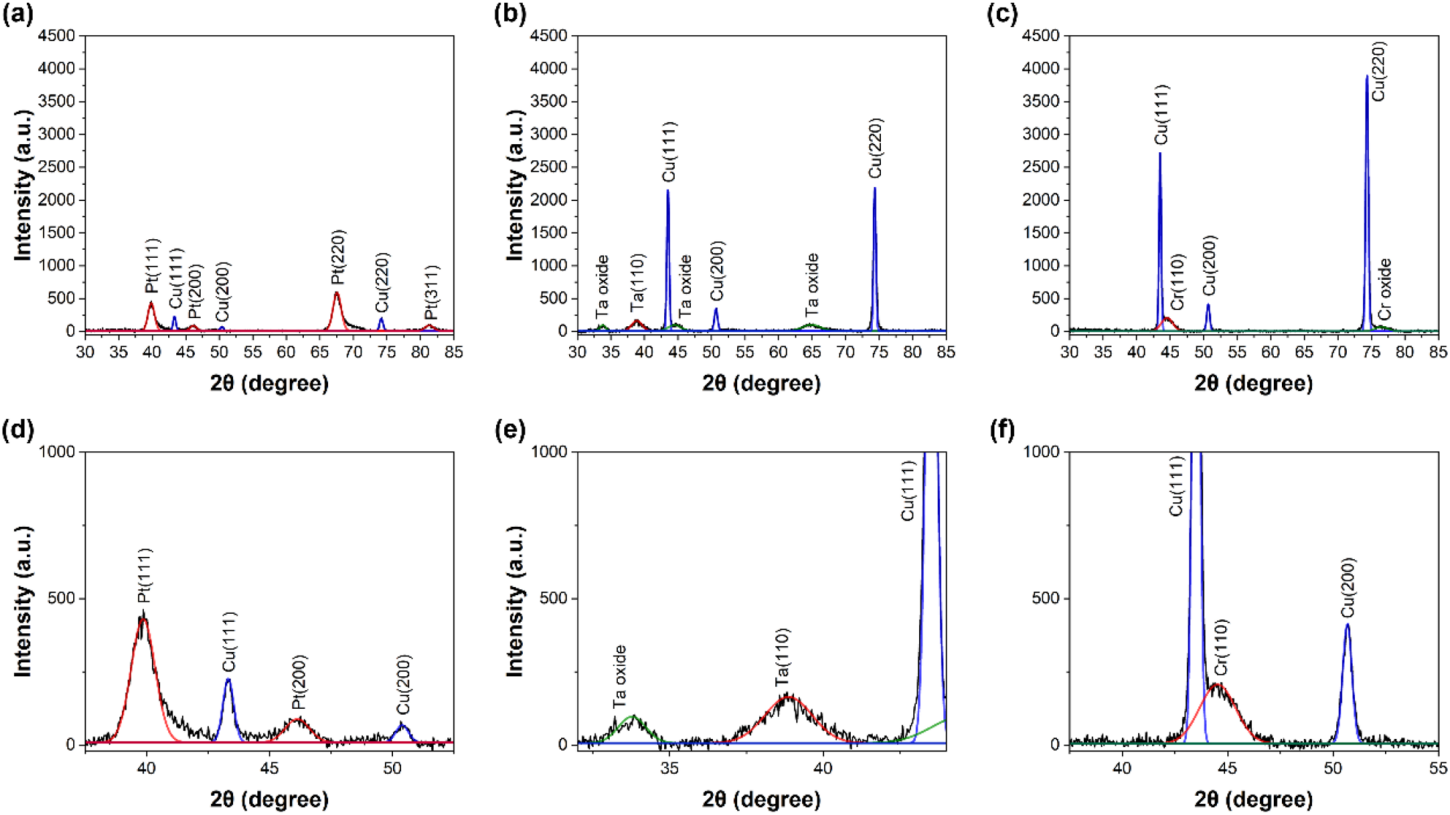
GIXRD graphs with crystal orientation indexing. (**a**) Pt, (**b**) Ta, (**c**) Cr passivated sample and enlarged size images (**d**) Pt, (**e**) Ta, (**f**) Cr passivated sample.

In addition to surface roughness and morphology confirmed by AFM, XRD was conducted to investigate the film characteristics. XRD is primarily employed as a suitable method for obtaining information related to grain size and crystallinity. The analysis was conducted on the metal passivation layers. To examine the passivation thin films at the nanoscale level, XRD analysis was conducted using GIXRD (Grazing Incidence X-ray Diffraction). Figure 6 shows GIXRD graphs analyzing the surfaces with the application of each metal passivation layer. Graph at Fig. 6a–c represent information for samples using Pt, Ta, and Cr passivation layers, respectively. The sample using Ti is excluded from the comparison as no peak was observed. GIXRD graphs of Ti are shown through Fig. S3a,b. In the graph for each sample, peaks corresponding to the respective metal and Cu peaks beneath the passivation layer can be observed.

Using the confirmed metal peaks, the size of the grains present in the metal passivation layers were calculated. The calculation of grain sizes (L) for the three types of metal passivations used the Scherrer equation and was represented by Eq. (1)^24^.

1 $$L=\frac{\mathrm{K \lambda }}{\mathrm{B Cos\theta }}$$

λ represents the wavelength of X-ray, K is the shape factor and B is full width at half maximum (FWHM). The values were obtained by fitting the XRD graph peaks. The fitted graphs are magnified and presented in Fig. 6d–f. The grain sizes of the Pt sample is confirmed to be 7.7 nm in the (111) plane, 7.8 nm in the (200), 7.1 nm in the (220) and 7.3 nm in the (311). Subsequently, for the Ta sample, the grain size was 4.9 nm in the (110) plane, and for the Cr sample, it was 4.5 nm in the (110) plane. The grain sizes obtained through GIXRD indicate in Table 2. In the graph of the sample with Pt, peaks representing various planes were observed and compared by averaging. A smaller grain size is advantageous for securing grain boundaries, and diffusion at lower temperatures is more favorable^19^. When comparing the grain sizes of metal passivation layers, the Pt sample exhibit larger grain size than the other metal passivation layers. Therefore, there is a possibility that the Pt sample may exhibit slower diffusion due to relatively fewer grain boundaries compared to the other metal samples. However, when compared with the previously introduced XPS depth profile, it shows a different trend from that of the grain size. Even though Cr has the smallest grain size, it is difficult to confirm significant diffusion. Therefore, it can be inferred that factors other than grain size also play a role in the diffusion behavior.

**Table 2:** Summarize grain size and crystallinity values.

	Pt	Ta	Cr
Grain size (nm)	7.5	4.9	4.5
Crystallinity—Cu (111)	5.29	0.33	0.40
Crystallinity—Cu (220)	5.15	0.32	0.22

Using GIXRD, it was possible to obtain information not only grain size but also crystallinity. The crystallinity was compared by analyzing the peak areas in the graphs of passivated metal samples. The atomic diffusion towards region of higher crystallinity is more pronounced than diffusion towards region of lower crystallinity^25^. Therefore, the comparison of crystallinity can serve as an effective factor for observing difference in diffusion behavior. The underlying Cu layer beneath the passivation metals is common and deposited under the same conditions. Therefore, comparison was made based on the Cu (111) and Cu (220). Numerical calculations for the comparison of crystallinity were conducted with the ratio of peak area of metal plane to peak area of Cu (111) and Cu (220)^26^. For the Pt sample, the crystallinity values for the (111), (200), (220), and (311) planes exhibit to be 4.3, 0.8, 7.3, and 1.1, respectively. In Ta and Cr samples, the crystallinity values are lower compared to the Pt sample, with values of 0.3 for the (110) in the case of Ta and 0.4 for the (110) in the case of Cr. The crystallinity comparison based on Cu (220) is shown in Table 2. For the Pt sample, due to the numerous peaks, the relative intensity ratios were used and it was calculated using the weighted average method. When setting the Pt (220) plane's intensity as 1, Pt (111) corresponds to approximately 0.94, Pt (200) to about 0.12, and Pt (311) to around 0.13. Upon comparing the values, it can be observed that the sample with Pt exhibits higher crystallinity compared to the samples with Ta and Cr. This result is consistent with the trend observed in the previously introduced XPS depth profile. In the XPS depth profile, it can be confirmed that atomic percentage of Cu diffused above Pt is significantly higher than that of Cr. Similarly, when comparing the values of crystallinity, the value of the Pt is about 13 and 23 times higher than that of Cr for the Cu (111) and Cu (220) orientation, respectively. Crystallinity based on Cu (111) and Cu (220) were compared between metals through a weighted average. And it was confirmed that the size of the crystalline value decreased in the order of Pt, Ta, and Cr, consistent with the amount of Cu diffusion confirmed in the XPS depth profile. As mentioned above, the diffusion behavior confirmed using XPS shows a trend that is almost similar to the crystallinity values rather than surface roughness and grain size. Therefore, in the selection of metal passivation material for low-temperature copper bonding, crystallinity can be considered as another crucial factor.

**Figure 7:**
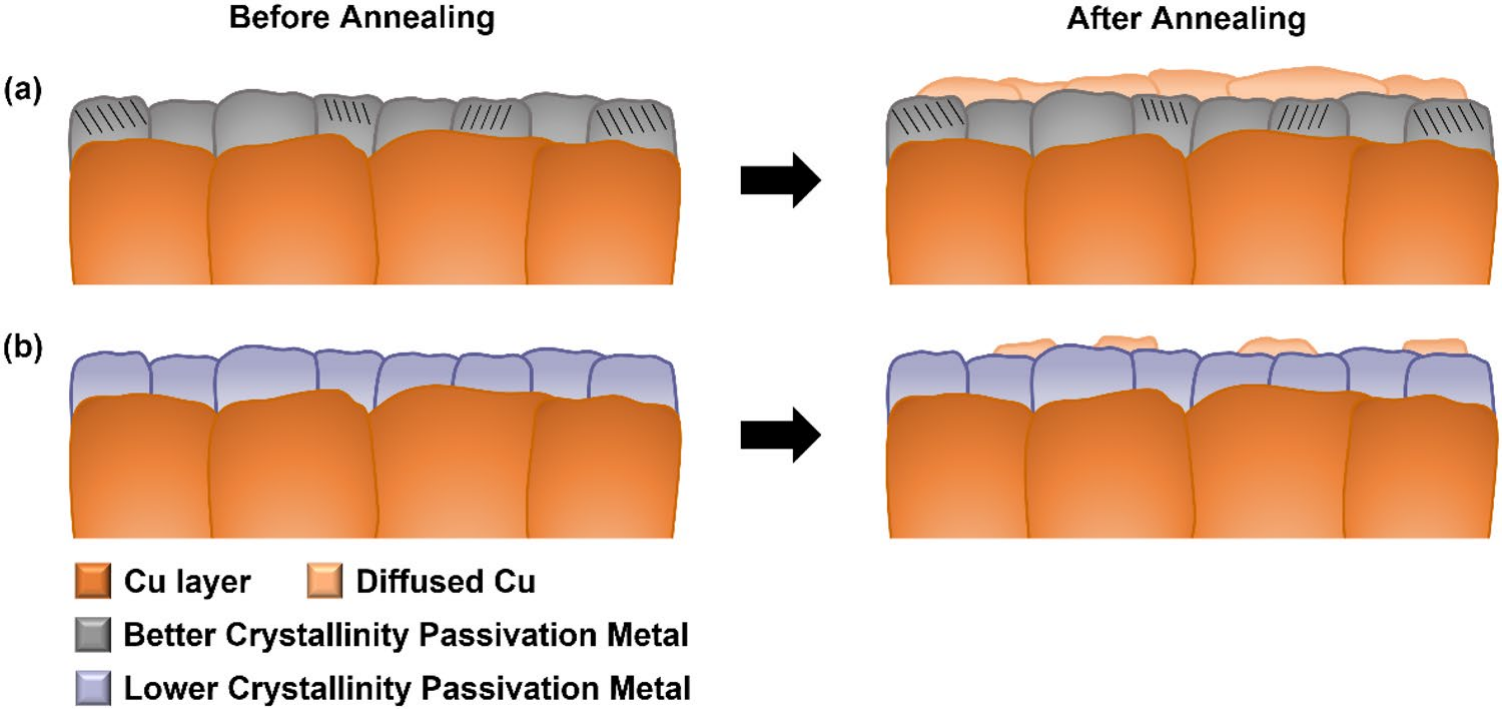
Schematic of Cu diffusion according to the crystallinity of passivation metal. (**a**) Pt sample with better crystallinity (**b**) relatively lower crystallinity of Cr sample.

Figure 7 is schematic illustrating the difference in Cu diffusion based on the passivation metals as described previously. Image in Fig. 7a represents the sample with using Pt passivation layer. When Pt passivation is applied, it exhibits relatively better crystallinity, showing better diffusion of Cu over Pt layer. In contrast, the image in Fig. 7b illustrates relatively lower crystallinity with Cr passivation, resulting in limited diffusion of Cu.

### Comparison of diffusion behavior through actual bonding

**Figure 8:**
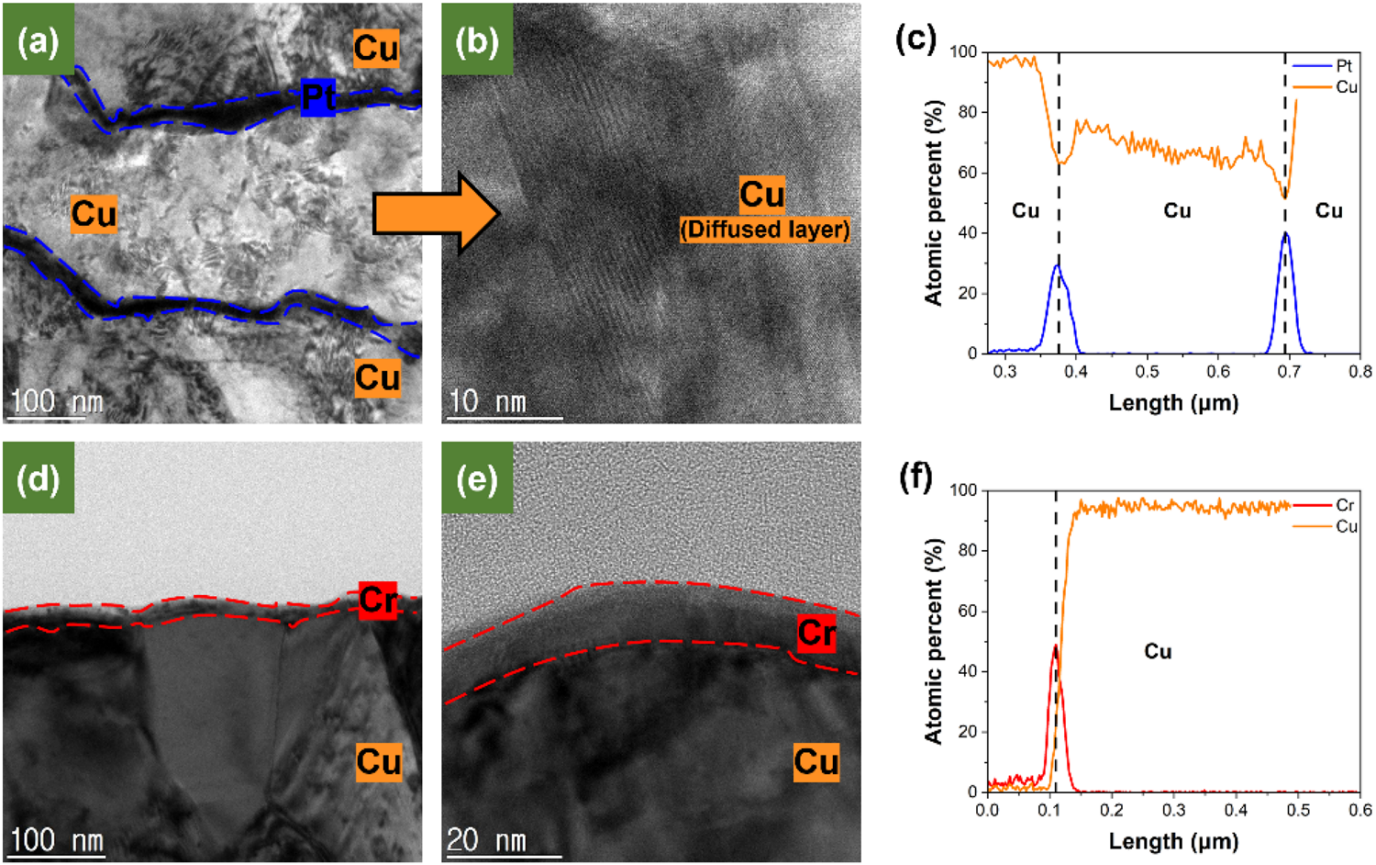
TEM images and EDS line scan (**a**) bonding interface at Pt sample, (**b**) diffused Cu layer at pt sample, (**c**) EDS line scan of Pt passivated bonding sample, (**d**) Surface of the Cr sample that failed to bonding, (**e**) Cr sample without diffused Cu, (**f**) EDS line scan of Cr passivated non-bonding sample. The samples were bonded under condition 200 °C, 30 min and 1 MPa.

Pt and Cr samples with different crystallinity were actually bonded using flip chip bonder and compared with the diffusion behavior confirmed using the XPS depth profile. TEM images in Fig. 8 show the diffused Cu at the interface of the bonded sample. Image at Fig. 8a represents the formation of a diffused Cu layer when bonding the Pt sample with a relatively high crystallinity. Continuing, Fig. 8b is an enlarged image of the diffused Cu layer. It shows that Cu diffused in state of amorphous and subsequently underwent recrystallization in accordance with the Cu bonding mechanism with passivation^27^. In the cases of Fig. 8d,e, the Cr sample with a low crystallinity did not bond under the same conditions as the Pt sample. Therefore, analysis was conducted on one side of the sample. It can be observed that there is almost no amount of diffused Cu on top of the Cr, confirming that Cu bonding did not take place. Graphs at Fig. 8c,f represent respective Line EDS (Energy Dispersive Spectrometer). In case of Fig. 8c, it can be observed that Cu has diffused between the Pt layers, forming a Cu layer. On the contrary, in case of Fig. 8f, it can be observed that there is almost no diffused Cu over the Cr. Through the TEM images, the bonding interfaces between Pt and Cr samples with different crystallinity were observed, and it was confirmed that it was consistent with the XPS diffusion behavior. Therefore, even in bonding conditions where temperature, pressure and surrounding environment influence exist, crystallinity acts as a crucial factor.

## Conclusions

As the performance of devices improves through 3D integration technology, Cu bonding becomes even more crucial. In this research, an analysis on the cause of diffusion behavior of metal passivation copper bonding was conducted for low-temperature copper bonding. Through the SAM images, different bonding interfaces could be identified under the same bonding conditions. The difference in diffusion behavior was confirmed with the XPS depth profile, and various analyses were conducted to examine the cause. It was verified that the roughness and grain size of the metal passivation layer did not match the tendency of diffusion behavior, while the crystallinity showed a tendency almost similar to that of diffusion behavior. The diffusion behavior of bonded Pt and Cr passivation samples with different levels of crystallinity was compared with the diffusion behavior of XPS depth profile, and the consistent trend was confirmed. Therefore, it was confirmed that crystallinity is one of the important diffusion factors in the copper bonding process using metal passivation. Metal passivation and inter-diffusion studies of copper are expected to contribute to the development of low-temperature copper bonding technology and further hybrid bonding.

### Supplementary Information

Supplementary Figures.

## Data Availability

The datasets used and/or analysed during the current study available from the corresponding author on reasonable request.
