# Supplementary material for: Unraveling diffusion behavior in Cu-to-Cu direct bonding with metal passivation layers
Source: Sci Rep. 2024 Mar 20;14:6665. doi: 10.1038/s41598-024-57379-2 (PMC10954753; doi:10.1038/s41598-024-57379-2)
Supplement: Supplementary file 1 — Supplementary Figures. [file 41598_2024_57379_MOESM1_ESM.docx]

**Supplementary Information**

**Unraveling Diffusion Behavior in Cu/SiO_2_ Hybrid Bonding with Metal Passivation Layers**

Min Seong Jeong, Sang Woo Park, Yeon Ju Kim, Ji Hoon Kim, Seul Ki Hong, Sarah Eunkyung Kim, Jong Kyung Park*

Department of Semiconductor Engineering, Seoul National University of Science and Technology, Seoul, Republic of Korea

* Corresponding to jkpark1@seoultech.ac.kr


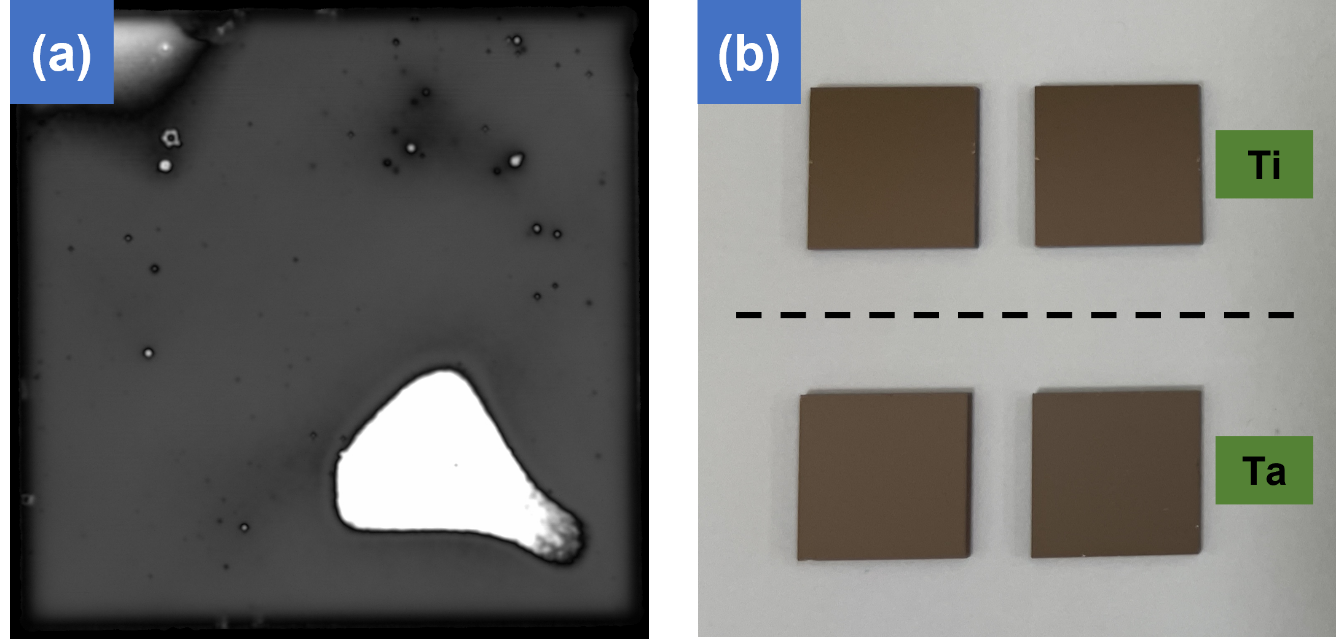


**Figure S1.** SAM image and photograph using metal passivation bonding samples. (a) SAM image with Pt passivation layer at 200℃, 30min and 1MPa condition. (b) Picture with Ti and Ta passivation layer. Ti sample was separated before the SAM analysis. And Ta sample was not bonded. Ti and Ta bonding samples were conducted same condition with pt sample. (200℃, 30min and 1MPa condition)


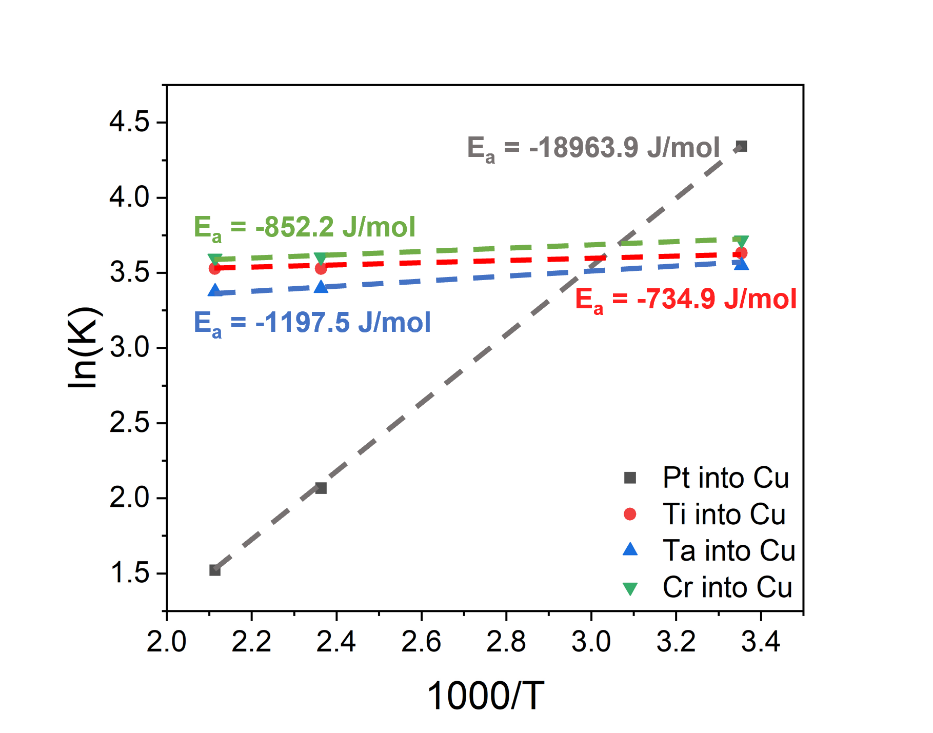


**Figure S2.** Graph for obtaining activation energies about various metal passivated samples. The activation energy of the Pt sample was the smallest compared to other samples. However, the activation energy values of other samples showed a negligible level of differences.


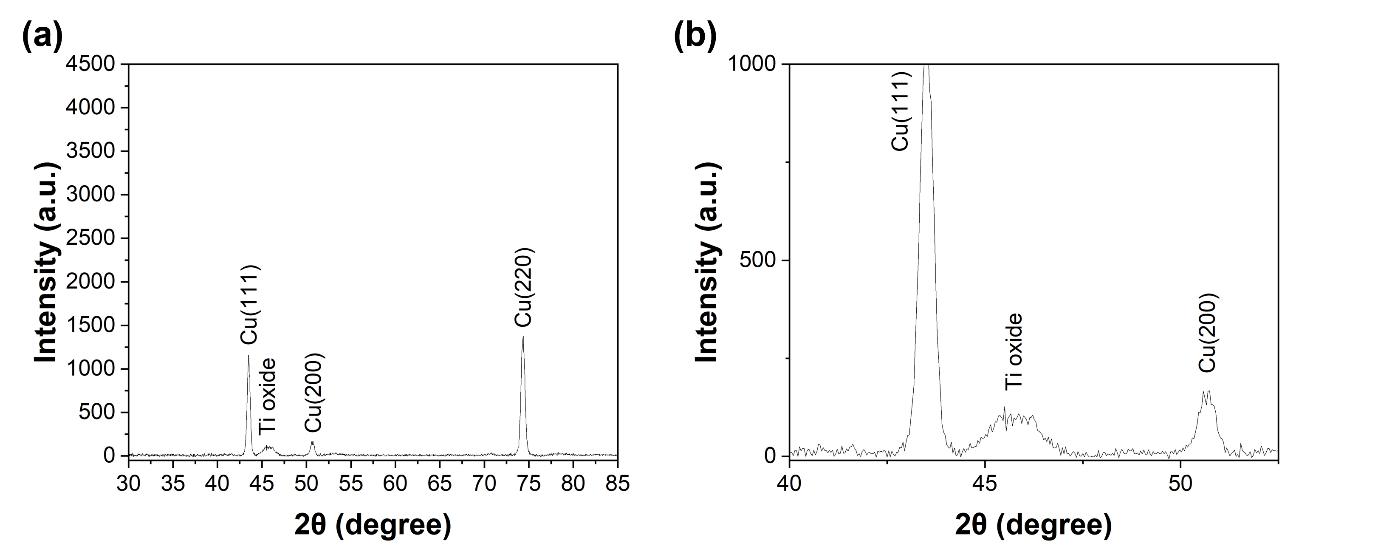


**Figure S3.** GIXRD graphs with crystal orientation indexing (a) Ti. And enlarged size image (b). No Ti peak suitable for confirming grain size and crystallinity has been identified.
